# Clinicopathological Characteristics, Prognosis, and Survival of HER2-Low Breast Cancer Patients Based on a Retrospective Cohort Study of 14,642 Patients

**DOI:** 10.3390/cancers18101637

**Published:** 2026-05-19

**Authors:** Wantong Sun, Xinyu Hou, Zihan Yang, Guozheng Li, Lei Zhang, Qin Wang, Xu He, Xin Zhang, Lei Liu, Changjun He, Shouping Xu

**Affiliations:** 1 Department of Breast Surgery, Harbin Medical University Cancer Hospital, Harbin 150040, China; sunwt0212@163.com (W.S.); 2024021727@hrbmu.edu.cn (X.H.); yangzihan728@sina.com (Z.Y.);; 2 Department of Thyroid Surgery, The Second Affiliated Hospital of Zhejiang University School of Medicine, Hangzhou 310009, China; 3 Heilongjiang Academy of Medical Sciences, Harbin 150086, China; 4 Key Laboratory of Tumor Biotherapy of Heilongjiang Province, Harbin Medical University Cancer Hospital, Harbin 150081, China; 5 Department of Pharmacology, College of Pharmacy of Harbin Medical University, The State-Province Key Laboratories of Biomedicine-Pharmaceutics of China, Harbin 150086, China; 6 Department of Thoracic Surgery, Harbin Medical University Cancer Hospital, Harbin 150040, China

**Keywords:** breast cancer, HER2-low, clinicopathological characteristics, prognosis, survival

## Abstract

Background and purpose: With the emergence of HER2 antibody–drug conjugates, new treatment options have been provided to patients with breast cancer, especially those with low HER2 expression. The biology of HER2-low breast cancer is not yet well understood. This study included the largest sample size thus far to explore the different clinicopathological characteristics, pathological complete responses (pCRs), and prognoses of HER2-low cancers compared with those of HER2-0 and HER2-high cancers. Methods: This study included 14,642 female breast cancer patients and 544 female patients with breast cancer from the TCGA. A logistic regression model was used to determine predictive factors for pCR. K–M curves and log-rank tests were applied to determine OS and DFS. Results: Compared with HER2-0 and HER2-high patients, the HER2-low group had a greater proportion of members in the N1–2 group and with grade I–II disease, a tumor size ≤ 20 mm, a Ki-67 index ≤ 14%, and invasive cancer. Among the HR+ subtypes of the HER2-low subgroup, the distribution of pathological types, pN stages, Ki-67 values, and tumor sizes significantly differed. In the triple-negative breast cancer subtypes of HER2-low patients, the distributions of age, pathological type, histological grade, and Ki-67 index were significantly different. The Ki-67 index and pathological type were independent factors that affected the pCR of the axillary lymph nodes. HER2 expression was mostly driven by patients who switched to or from HER2-low expression after neoadjuvant chemotherapy. The pCR rate and survival did not differ markedly across the three HER2 subtypes. Conclusions: HER2-low breast cancer is distinct from HER2-0 and HER2-high breast cancer with respect to clinicopathological characteristics. HER2-low breast cancer is highly unstable during chemotherapy; therefore, reevaluating HER2 expression may provide new treatment strategies for some patients after neoadjuvant therapy.

## Simple Summary

HER2-low breast cancer has become clinically important because newer antibody–drug conjugates may benefit patients whose tumors do not meet the criteria for HER2-positive disease. In this retrospective study, we analyzed 14,642 breast cancer patients, including 1526 patients who received neoadjuvant chemotherapy, and combined these findings with survival data from the TCGA database. HER2-low tumors had distinct clinicopathological features, and HER2 expression frequently changed after neoadjuvant chemotherapy. However, HER2-low status was not associated with significantly different pathological complete response rates or survival outcomes. These findings help clarify the clinical behavior of HER2-low breast cancer in a large Chinese cohort.

## 1. Introduction

Breast cancer (BC) is a highly heterogeneous group of malignant tumors. BC can be divided into different molecular subtypes through the expression of hormone receptors (HRs) and human epidermal growth factor receptor 2 (HER-2), which also determine the basic treatment strategy for BC [1,2]. HER2 is an important prognostic and predictive marker [3]. HER2 status was defined in accordance with the 2018 American Society of Clinical Oncology/College of American Pathologists (ASCO/CAP) guidelines using immunohistochemistry (IHC) and/or in situ hybridization (ISH) assays [4]. HER2-enriched BC accounts for approximately 15–20% of all BCs and presents with clinical features and a poor prognosis [5,6]. Nevertheless, owing to agents targeting the HER2 pathway, when trastuzumab was first approved to treat HER2-enriched BC, prognosis significantly improved [7,8]. HER2 is clearly a key driver gene and therapeutic target in breast cancer, and its expression status directly affects the choice of targeted treatment strategy.

In addition to HER2-0 and HER2-high BC, when HER2 expression is IHC1+ or IHC 2+/ISH-negative, it is referred to as HER2-low BC, which comprises as much as 45–60% of HER2-0 BC cases [9,10]. Several clinical studies have demonstrated that trastuzumab and pertuzumab do not benefit HER2-low tumor patients [11,12]. However, antibody–drug conjugates (ADCs), such as trastuzumab–deruxtecan [13] and trastuzumab–duocarmazine [14], have achieved better efficacy and have approved indications for HER2-low BC.

On the basis of these results, HER2-low BC is now considered a distinct entity. The clinicopathological characteristics and predictive prognostic implications of HER2-low BC patients are inconsistent with previously reported results [15,16]. In recent years, HER2-low breast cancer has garnered significant attention because of the marked efficacy demonstrated by novel ADCs; however, the real-world clinical and pathological characteristics, chemotherapy response, and biological stability of this subtype remain unclear. Previous studies have been limited by small sample sizes and inconsistent findings, and large-scale data from China are lacking.

In summary, controversy remains regarding the clinicopathological features, chemotherapy sensitivity, and prognosis of patients with HER2-low-expressing breast cancer, and large-sample data from the Chinese population are lacking. This study examined the largest sample size thus far and aimed to comprehensively describe the clinicopathological characteristics, prognosis and response to neoadjuvant chemotherapy of HER2-low breast cancer patients. A large retrospective analysis of 14,642 cases revealed that HER2-low breast cancer has unique clinical and pathological characteristics and that HER2 status is highly unstable following chemotherapy; however, HER2 status does not influence the rate of pathological complete response (pCR) to neoadjuvant chemotherapy or survival. The aim of this study was to clarify the clinicopathological characteristics, chemotherapy responses, and dynamic changes in the HER2 status of HER2-low-expressing breast cancer patients. The results of this study provide real-world evidence for personalized clinical treatment of HER2-low-expressing breast cancer.

## 2. Materials and Methods

### 2.1. Patients

This study included 14,642 female breast cancer patients who underwent breast cancer surgery at Harbin Medical University Cancer Hospital between 1 January 2000 and 31 December 2022, as well as 1526 patients who received neoadjuvant therapy and surgery. The ethics committee of Harbin Medical University Cancer Hospital approved this study. All patients signed informed consent forms before they underwent treatment. A total of 544 female breast cancer patients from the TCGA database whose survival data were available were also included in this study.

The inclusion criteria were as follows: (i) received complete neoadjuvant chemotherapy (NACT) based on anthracycline drugs and paclitaxel; (ii) received curative surgery after NACT; and (iii) did not receive antitumor treatment before NACT. Notably, because of the financial burden, none of the patients included in our analysis received anti-HER2 therapy.

**Figure 1:**
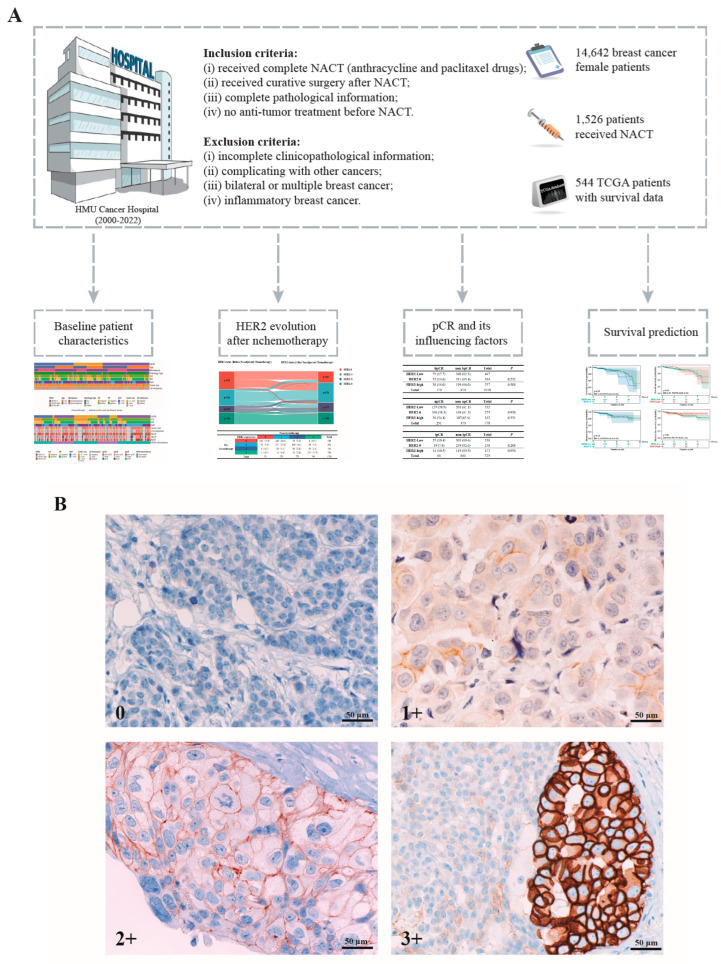
Study flowchart and immunohistochemistry images. (**A**) Flow diagram of patient selection. (**B**) Classification of breast cancers based on immunohistology staining. The IHC staining was performed according to standard procedures.

The exclusion criteria were as follows: (i) incomplete clinicopathological information; (ii) complicated by other cancers; (iii) bilateral or multiple breast cancer; and (iv) inflammatory breast cancer. The details are shown in Figure 1.

### 2.2. Clinicopathological Indicators

The levels of estrogen receptor (ER), progesterone receptor (PR), and Ki-67 were determined using IHC, and HER2 status was determined by IHC or fluorescent in situ hybridization (FISH). All ER, PR, and HER2 IHC and HER2 FISH assays were performed and officially reported by the Department of Pathology at our institution according to standard clinical protocols. An ER or PR level ≥ 1% was defined as ER- or PR-positive. If both ER and PR were positive, the case was defined as HR-positive. HER2 status was assessed in accordance with the 2018 ASCO/CAP guidelines [4]. HER2-low status was defined as IHC-1+ and IHC-2+ with negative ISH results. IHC-0 and IHC-3+ are referred to as HER2-0 and HER2-high, respectively.

### 2.3. Efficacy Assessments

A pathological complete response of the breast (bpCR) was defined as the absence of residual invasive cancer in the breast (although ductal carcinoma in situ (DCIS) was permitted). Axillary lymph node pCR (npCR) was defined as the absence of evidence of disease in the axillary lymph nodes. Total pCR (tpCR) was required to meet the conditions of both bpCR and npCR.

### 2.4. Follow-Up

Overall survival (OS) was defined as the time from the date of primary breast cancer diagnosis to the date of death or last follow-up. Disease-free survival (DFS) refers to the duration between the date of surgery and the date of relapse or death due to any factor. The survival data were only collected from the TCGA database.

### 2.5. Statistical Analysis

Comparisons of clinical characteristics between groups were performed using the chi-square test or Fisher’s exact test. Univariate and multivariate logistic regression analyses were performed to explore the predictive factors for pCR. Multivariate logistic regression included variables with a *p* value < 0.1 in the univariate analysis, and missing values were handled using the complete-case approach. The model showed good fit as assessed by the Hosmer–Lemeshow test. The Kaplan–Meier method was used to estimate survival curves, and the log-rank test was used to test for differences across groups. All the data were analyzed using IBS SPSS (version 22.0). All tests were two-sided, and *p* values < 0.05 were considered to indicate statistical significance.

## 3. Results

### 3.1. Clinicopathological Characteristics Based on HER2 Status

In total, 14,642 patients were included in the analysis, and an additional 1526 patients received neoadjuvant chemotherapy (NACT) with anthracycline and paclitaxel (Figure 1A). To clarify the expression levels of HER2 for different statuses, representative immunohistological staining images of HER2 expression are provided in Figure 1B. Among the 6575 (44.9%) patients who had HER2-low status, 4423 (30.2%) were classified as HER2-0, and 3644 (24.9%) were classified as HER2-high (Table 1).

**Figure 2:**
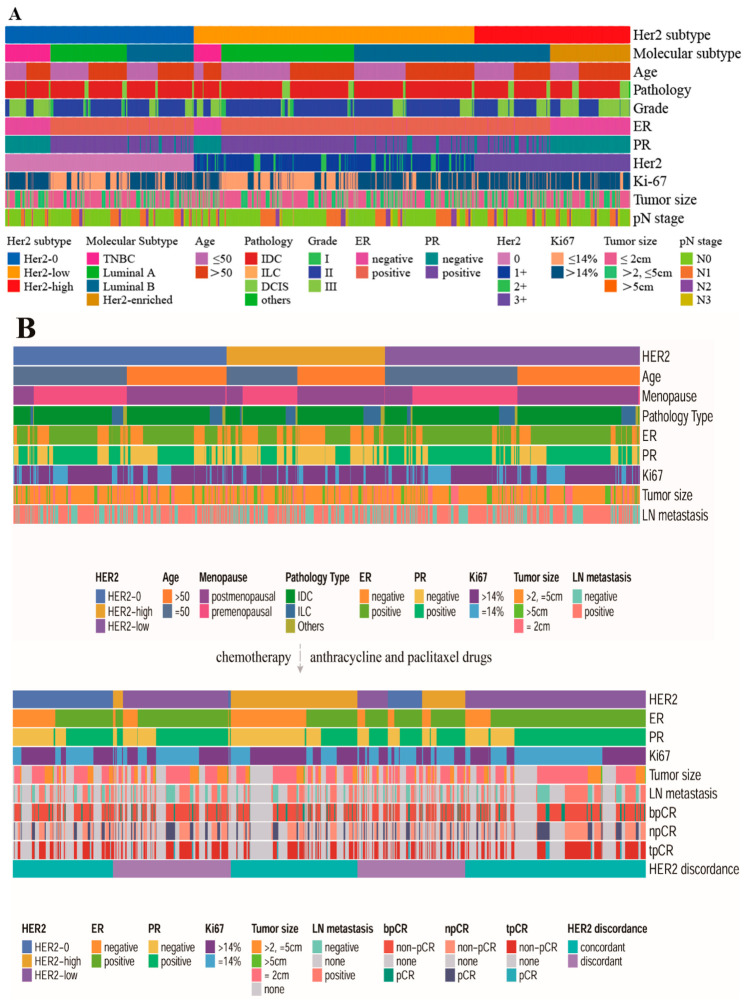
The heatmap of baseline. (**A**) The heatmap of 14,642 patients’ baseline. (**B**) The heatmap of 1526 patients’ baseline.

Compared with HER2-0 patients, greater proportions of HER2-low patients were in the N1–N3 group (38.0%) and had invasive cancer (IDC + ILC) (92.8%), ER-positive status (89.7%), PR-positive status (84.0%), HR-positive status (90.3%) and Ki-67 values ≤ 14% (44.9%). Compared with HER2-high patients, a greater proportion of HER2-low patients were ≤50 years of age (46.4%) and had N1–N2 status (33.9%), a tumor size ≤ 20 mm (55.7%), invasive cancer (IDC+ILC) (92.8%), ER-positive status (89.7%), PR-positive status (84.0%), HR-positive status (90.3%) and Ki-67 values ≤ 14% (44.9%) (Table 1). The basic features of the 14,642 patients are shown in Figure 2A.

In general, more HER2-0 and HER2-low patients had a Ki-67 value ≤ 14%, a lower pN stage, and a smaller tumor size. In contrast, HER2-high patients had the highest percentage with a Ki-67 index > 14%, N3 stage, and a large tumor size (Supplementary Figure S1A). Considering that the highest proportion of patients with ER-/PR-positive status were HER2-low patients, this study describes the proportion of HER2-low patients with different ER/PR statuses. As the expression levels of ER and PR increased, the percentage of patients with HER2-low status gradually increased (Supplementary Figure S1B,C).

Owing to the significant difference in malignancy between invasive cancer (IDC+ILC) and DCIS, the clinicopathological characteristics of invasive breast cancer and DCIS with different HER2 statuses were explored. Among the 13,208 invasive breast cancer patients, compared with HER2-0 patients, HER2-low patients were more likely to have grade II disease (81.0% vs. 69.6%), N1–3 stage disease (40.90% vs. 36.5%), ER-positive status (89.8% vs. 73.8%), PR-positive status (84.0% vs. 69.2%) and Ki-67 values ≤ 14% (42.8% vs. 37.9%). Compared with HER2-high patients, HER2-low patients presented similar trends in ER-positivity (89.8% vs. 49.0%), PR-positivity (84.0% vs. 38.9%) and Ki-67 values ≤ 14% (42.8% vs. 9.9%), and other characteristics, including age ≤ 50 years (45.8% vs. 43.2%), grade I and II disease (84.8% vs. 58.7%), N0 status (59.1% vs. 55.8%), and tumor size ≤ 20 mm (55.1% vs. 44.1%), were significantly different (Supplementary Table S1).

Among the 1366 DCIS patients, compared with HER2-0 patients, HER2-low patients were more likely to have grade III disease (40.1% vs. 33.4%), and significant differences in other characteristics were not observed. Compared with HER2-high patients, HER2-low patients presented similar trends in terms of the incidence of invasive HER2-low and HER2-high cancers (Supplementary Table S2).

### 3.2. Clinicopathological Characteristics of the HR+ and TNBC Subtypes

In all the populations, most of the patients (45%) had HER-2 low status, while 30% of the patients had HER2-0 status and 25% had HER2-high status. Regardless of HER-0 or HER-low status, HR+ patients accounted for a greater proportion (76% and 91%, respectively) of all patients (Supplementary Figure S2A). Among the HR+ patients, 54% had HER-2 low status. Among the TNBC patients, 38% had HER-2 low status (Supplementary Figure S2B).

We further compared the characteristics of the HR+ and TNBC subtypes, namely, HER2-0, HER2-low, and HER2-high. In terms of the HR+ subtypes, compared with HER2-0 patients, HER2-low patients accounted for a greater proportion of patients with invasive cancer (IDC+ILC) (92.9%), N1–3 disease (38.8%), a Ki-67 index > 14% (52.0%) and tumors > 20 mm in size (43.3%). Compared with HER2-high patients, HER2-low patients comprised a greater proportion > 50 years (52.3%) and with a Ki-67 index ≤ 14% (48.0%), a tumor size ≤ 20 mm (56.7%), invasive cancer (IDC+ILC) (92.9%) and N1 status (25.5%) (Table 2). More intuitively, the statistical analysis revealed that the proportion of HER2-0 and HER2-low patients increased with increasing Ki-67 values and tumor size, and the proportion of HER2-high patients increased with increasing Ki-67 values, tumor size, and pN stage among the HR+ subtypes (Supplementary Figure S3).

Among the 10,136 HR+ invasive breast cancer patients, compared with HER2-0 patients, HER2-low patients were more likely to have N1–3 disease (41.7% vs. 39.0%), a Ki-67 index > 14% (54.0% vs. 51.5%) and a tumor size > 20 mm (44.0% vs. 39.7%). Compared with HER2-high patients, HER2-low patients were more likely to have an age > 50 (53.1% vs. 48.9%), grade I or II disease (89.6% vs. 69.1%), N0 disease (58.3% vs. 56.0%), a Ki-67 index ≤ 14% (46.0% vs. 11.9%) and a tumor size ≤ 20 mm (56.1% vs. 44.8%) (Supplementary Table S3). Among the 917 DCIS patients, compared with HER2-0 patients, HER2-low patients were more likely to have grade II or III disease (68.0% vs. 52.7%), and the other characteristics were not significantly different. Compared with HER2-high patients, HER2-low patients presented a similar trend to that observed when HER2-low and HER2-high HR+ invasive breast cancer patients were compared, except for age and pN stage (Supplementary Table S4).

In terms of the TNBC subtypes, compared with HER2-0 patients, a larger proportion of HER2-low patients were aged > 50 years (65.1%) and had DCIS or other BC types (7.9%) and a Ki-67 index ≤ 14% (16.1%) (Table 3). The Ki-67 results were similar to those for all the other types of breast cancer (55.1% vs. 59.4%) (Table 1) but contrary to the results for the HR+ subgroup (52.0% vs. 48.7%) (Table 2). Overall, compared with HER2-0 patients, HER2-low patients accounted for a greater percentage of patients with a Ki-67 index ≤ 14% (59% vs. 41%) in the TNBC cohort (Supplementary Figure S3A). Owing to the small number of DCIS samples in the TNBC group, this study did not conduct a subgroup analysis of invasive cancer and DCIS among TNBC patients.

### 3.3. Baseline Patient Characteristics of the IHC1+ and IHC2+ Subgroups in the HER2-Low Cohort

Among HER2-low breast cancer patients, a higher percentage of IHC-2+ patients had invasive cancer (IDC+ILC) (99%), a Ki-67 index > 14% (65.5%) and a tumor size > 20 mm (48.1%) (Supplementary Table S5). Among patients with the HR+ phenotype, IHC-2+ patients had a higher percentage of invasive cancer (99.1% vs. 91.6%), a Ki-67 index > 14% (63.2% vs. 49.6%) and a tumor size > 20 mm (47.8% vs. 42.3%) (Supplementary Table S6). Among patients with a TNBC phenotype, patients who were IHC-2+ also had a higher percentage of invasive cancer (98.3% vs. 90.6%, *p* = 0.016), an N1 stage (25.9% vs. 18.7%) and an N3 stage (6.9% vs. 3.1%) (Supplementary Table S7).

### 3.4. HER2 Evolution from Pre- to Post-Neoadjuvant Chemotherapy

**Figure 3:**
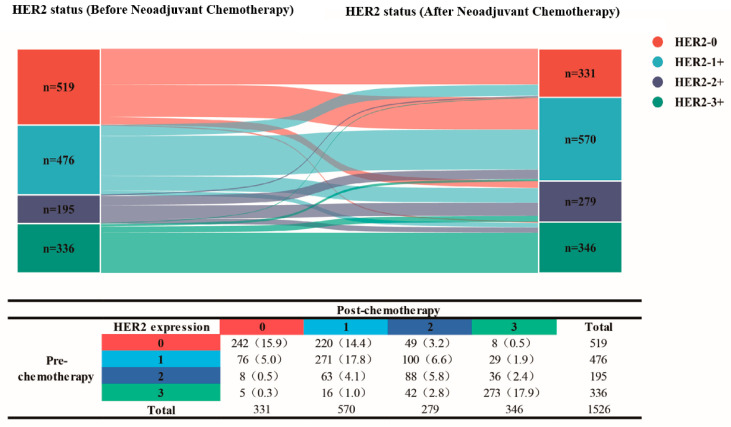
The evolution of HER2 expression from pre- to post-chemotherapy. Absolute numbers and percentages are reported.

The baseline data of 1526 patients who received neoadjuvant chemotherapy are shown in Figure 2B. The evolution of HER2 expression from pre- to post-neoadjuvant chemotherapy is shown in Figure 3. The overall rate of HER2 discordance was 42.6% (*n* = 652). The main clinicopathological features according to HER2 discordance status are shown in Supplementary Table S8.

The rate of HER2 discordance was mostly driven by patients who switched to or from HER2-low expression. In particular, in 14.4% of the patients, a conversion from the HER2-0 to the HER2-1+ phenotype was observed. The changes from pre-neoadjuvant chemotherapy HER2-1+ status to post-neoadjuvant chemotherapy HER2-0 and HER2-2+ status accounted for 5.0% and 6.6% of patients, respectively. HER2-2+ patients mostly transformed into HER2-1+ status (63, 4.1%) or maintained HER2+ status (88, 5.8%) after NACT.

The HER2-3+ phenotype was most stable in both the pre-neoadjuvant chemotherapy and post-neoadjuvant chemotherapy groups, with 4.1% of the total patients exhibiting loss of HER2 expression. Among patients with HER2 loss who had HER2-3+ breast cancer, the great majority (n = 58, 3.8%) exhibited a HER2-low phenotype.

### 3.5. Predictive Value of HER2 Status for Chemotherapy Response and Survival Prediction

The pCR rates of HER2-low breast cancer were 17.7% (bpCR), 38.9% (npCR), and 10.4% (tpCR). HER2 status was not associated with the rates of bpCR, npCR, or tpCR (Supplementary Table S9). Moreover, in the HR+ and TNBC subtypes, significant differences in the pCR rate among the HER2-0, HER2-low, and HER2-high subtypes were not observed (Supplementary Table S10). The results of this study summarize the studies on HER2-low breast cancer, which compared the differences in pCR between HER2-low and HER2-0 tumors (Supplementary Table S11). These results varied substantially. However, multiple studies have confirmed that the pCR rate of HER-low patients is significantly greater than that of HER2-0 patients.

**Figure 4:**
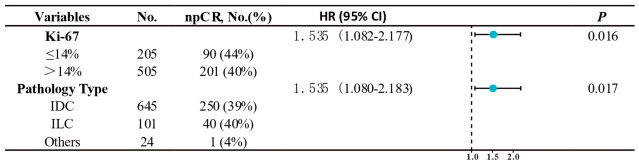
Multivariate analysis of npCR in the neoadjuvant chemotherapy patients.

The predictive factors affecting pCR were also detected through univariate square analysis and multivariate logistic regression analysis. The results revealed that the rate of npCR was influenced by multiple factors, including the Ki-67 index (43.9% vs. 35.6%) and pathological type (4.2% vs. 38.8% and 39.6%) (Supplementary Table S12). The Ki-67 index (HR = 1.535; 95% CI 1.082–2.177; *p* = 0.016) and pathological type (HR = 1.535; 95% CI 1.080–2.183; *p* = 0.017) were also independent risk factors for the rate of npCR (Figure 4). Only tumor size was correlated with bpCR and tpCR (Supplementary Tables S13 and S14). Notably, the results of the regression analysis revealed that HER2 status does not affect the pCR of each HER2 subtype.

We also evaluated the survival of patients with various subtypes of HER2 using the TCGA database, and the results revealed no significant difference in OS or DFS between HER2-low and HER2–0/HER2-high patients (Figure S4). We also summarized studies on the survival of patients with HER2-low breast cancer compared with those with HER2–0 tumors (Supplementary Table S15). Most research results have shown that there is no difference in survival between HER2–0 and HER2-low patients.

## 4. Discussion

With the continuous progress of precision treatment for BC, HER2-low BC has also attracted increasing attention. Here, a retrospective analysis of the largest sample (14,642 patients) thus far was conducted to compare the clinicopathological characteristics and the different responses to NACT among HER2-0, HER2-low, and HER2-high BC patients.

Patients with HER2-low BC had significantly different clinicopathological characteristics from those with HER2-0 and HER2-high BC and more favorable prognostic factors, such as the highest ratio in the Ki-67 index ≤ 14% and N1 groups. Compared with HER2–0 and HER2-high BC cases, HR+ BC accounted for the greatest proportion of HER2-low BC cases (90.3% vs. 76.1% and 48.8%, respectively). This finding is similar to previous research results [9,17].

This study focused on describing the clinicopathological characteristics of patients with different HER2 statuses and various subgroups (HR+ vs. TNBC and invasive breast cancer vs. DCIS). Few studies have explored the difference between HER2-low and HER2-high patients. Our results revealed that a higher proportion of HER2-low patients had N1 or N2 disease (33.9%, Table 1), grade I or II disease (84.8%, Supplementary Table S1; 89.6%, Supplementary Table S3), tumors ≤ 20 mm (55.7%, 56.7%, Table 1 and Table 2; 55.1%, 64.3%, 56.1%, 66.7%, Supplementary Tables S1–S4), a Ki-67 index ≤ 14% (44.9%, 48.0%, Table 1 and Table 2; 42.8%, 73.8%, 46.0%, 76.5%, Supplementary Tables S1–S4), and invasive cancer (92.8%, Table 1; 92.9%, Table 2).

Currently, multiple studies focus on comparing HER2-0 and HER2-low patients. In terms of pN stage and histological grade, previous studies have shown that compared with HER2–0 patients, HER2-low patients are more likely to have a higher pN stage [18,19] and a lower histological grade [19,20]. Studies have also shown no significant differences in the proportion of pN stages between HER2-0 and HER2-low patients [20,21]. Our study demonstrated that compared with HER2-0 patients, HER2-low patients had a higher pN stage (N1–3, 38%; Table 1; N1–3, 38.3%; Table 2; N1–3, 40.9%; Supplementary Table S1) but also had a higher histological grade (high grade, 40.1%; Supplementary Table S2; medium and high grade, 68.0%; Supplementary Table S4). These results were also similar in the subgroup analysis (HR+ vs. TNBC). In terms of tumor size, studies have shown no significant difference [19,22]. Our results are similar (Table 1 and Table 3 and Supplementary Tables S1, S2 and S4). However, in the HR+ subgroup, compared with the HER2-0 subgroup, the HER2-low subgroup had a greater percentage of tumors > 20 mm in size (43.3%, Table 2; 44.0%, Supplementary Table S3). In terms of pathological type, our study revealed that the HER2-low subgroup had a greater percentage of invasive cancer (92.8%, Table 1; 92.9%, Table 2). Except for the TNBC subtype, HER2-low is characterized mainly by DCIS (7.0%, Table 3), unlike the HER2-0 subtype. Previous studies have focused mostly on invasive cancer and have not described this characteristic.

The discordance in HER2 expression between pre- and post-chemotherapy was significant. Some studies have shown that resistance to tumor therapy, including endocrine therapy [23], chemotherapy [24], and anti-HER2 therapy [25], can lead to changes in HER2 status. Moreover, in early and late breast cancer [26], primary breast cancer and recurrent breast cancer [27] show changes in HER2 status. Interestingly, this change occurs mainly in the HER2-low subtype. A total of 51.8% of HER2-0 subtypes transformed to HER2-low subtypes. A total of 77.8% of the HER2-low subtypes remained in the HER2-low state. These findings may provide new insights into HER2-low breast cancer treatment.

With respect to the impact of HER2-low status on the response to NACT, the differences in pCR between HER2-low and HER2-0 tumors varied substantially across studies. To date, sixteen studies have investigated the role of HER2-low expression in the pathological response and survival after NACT (Figure 4). Among them, eight studies reported that the pCR rate of HER2-low patients was significantly lower than that of HER2-0 patients, but only three studies reported better survival [15,18,20,22,28,29,30,31]. The results of four of the eight studies were the same for the HR+ subtypes. Only Guansheng Zhong et al. demonstrated that the pCR rate of HER2-low patients is significantly lower than that of HER2-0 patients (46.6% vs. 48.8%, *p* < 0.001) by using a national cancer database. In the other six studies, no differences in pCR rates were detected [32,33,34,35,36]. Most studies have shown that there is no significant difference in survival between HER2-0 patients and HER2-low patients after NACT. Another five studies reported survival without the use of NACT [19,21,37,38,39]. Two studies revealed that HER2-low patients had better survival (DFS, *p* = 0.04; OS, *p* < 0.001; DFS, *p* < 0.001). The subgroup analysis in two studies, as well as that in other studies, revealed no significant difference in survival, which is consistent with our TCGA prediction results.

However, most of the studies are limited to patients in Western countries, and the largest study was carried out in China and included 3070 patients receiving NACT [40]. In contrast to other studies, the HER2-0 subtype had a higher pCR rate than the HER2-low subtype among HR+ subtypes (17.3% vs. 13.7%, *p* = 0.016), and there was no significant difference in survival (OS, *p* = 0.037; DFS, *p* = 0.610). Another study of 690 Chinese patients revealed that the pCR rate of HER2-low patients was slightly lower than that of HER2-0 patients (14.2% vs. 23.0%, *p* = 0.005), and DFS was not significantly different (*p* = 0.846). Therefore, further research is needed to evaluate pCR and survival in HER2-low patients in the Chinese population. Our study validated and added to this work on the basis of a larger sample size in China.

Several limitations exist in the current study. First, this is a retrospective study and is inevitably subject to selection bias and information bias. Second, complete survival data were not obtained, making it impossible to evaluate the survival of chemotherapy patients because of the relatively short follow-up time and limited incidence of death events. In addition, Ki-67 testing is subject to interlaboratory variability, and the results should be interpreted with caution. The findings of this study are observational and hypothesis-generating; the conclusions must still be validated by prospective studies.

## 5. Conclusions

In conclusion, this is the largest retrospective study in which the characteristics of patients with different HER2 statuses were described. The HER2-low subgroup had greater proportions of patients with N1–N3 disease, invasive cancer (IDC+ILC), ER-positive status, PR-positive status, HR-positive status and a Ki-67 index ≤ 14%. Moreover, HER2 status does not affect the pCR rate of neoadjuvant chemotherapy or survival in breast cancer patients. We also reported that the rate of HER2 discordance was mostly driven by patients who switched to or from HER2-low expression after neoadjuvant chemotherapy. These findings provide a reference basis for HER2-targeted treatment of HER2-low breast cancer. We will further supplement the survival information of patients and expand the sample size of patients receiving neoadjuvant chemotherapy, providing stronger evidence for the treatment response and survival of patients with different HER2 states.

## Figures and Tables

**Table 1:** Characteristics of all patients with HER2-low, HER2-high or HER2-0 breast cancer.

Variables	HER2-Low	HER2-0	*p*	HER2-High	*p*
	6575	4423		3644	
Age					
≤50	3052 (46.4)	2112 (47.8)	0.176	1605 (44.0)	0.022
>50	3523 (53.6)	2311 (52.2)		2039 (56.0)	
Pathology Type					
IDC+ILC	6100 (92.8)	4064 (91.9)	0.001	3044 (83.5)	<0.001
DCIS	454 (6.9)	323 (7.3)		589 (16.2)	
Others	21 (0.3)	36 (0.8)		11 (0.3)	
pN stage					
N0	4074 (62.0)	2933 (66.3)	<0.001	2295 (63.0)	<0.001
N1	1639 (24.9)	982 (22.2)		785 (21.5)	
N2	590 (9.0)	356 (8.0)		323 (8.9)	
N3	272 (4.1)	152 (3.4)		241 (6.6)	
ER status					
Negative	676 (10.3)	1117 (25.3)	<0.001	1950 (53.5)	<0.001
Positive	5899 (89.7)	3306 (74.7)		1694 (46.5)	
PR status					
Negative	1055 (16.0)	1325 (30.0)	<0.001	2323 (63.7)	<0.001
Positive	5520 (84.0)	3098 (70.0)		1321 (36.3)	
HR status					
Negative	639 (9.7)	1059 (23.9)	<0.001	1864 (51.2)	<0.001
Positive	5936 (90.3)	3364 (76.1)		1780 (48.8)	
Ki-67					
≤14%	2955 (44.9)	1797 (40.6)	<0.001	473 (13.0)	<0.001
>14%	3620 (55.1)	2626 (59.4)		3171 (87.0)	
Tumor size					
≤20 mm	3661 (55.7)	2533 (57.3)	0.231	1647 (45.2)	<0.001
20–50 mm	2827 (43.0)	1829 (41.4)		1910 (52.4)	
>50 mm	87 (1.3)	61 (1.4)		87 (2.4)	

**Table 2:** Characteristics of HR+ patients with three HER2 breast cancer subtypes.

Variables	HER2-Low	HER2-0	*p*	HER2-High	*p*
	5936	3364		1780	
Age					
≤50	2829 (47.7)	1620 (48.2)	0.659	929 (52.2)	0.001
>50	3107 (52.3)	1744 (51.8)		851 (47.8)	
Pathology Type					
IDC+ILC	5512 (92.9)	3058 (90.9)	0.003	1566 (88.0)	<0.001
DCIS	409 (6.9)	294 (8.7)		214 (12.0)	
Others	15 (0.3)	12 (0.4)		0 (0.0)	
pN stage					
N0	3634 (61.2)	2166 (64.4)	0.008	1091 (61.3)	0.018
N1	1511 (25.5)	791 (23.5)		421 (23.7)	
N2	543 (9.1)	298 (8.9)		164 (9.2)	
N3	248 (4.2)	109 (3.2)		104 (5.8)	
Ki-67					
≤14%	2852 (48.0)	1725 (51.3)	0.003	253 (14.2)	<0.001
>14%	3084 (52.0)	1639 (48.7)		1527 (85.8)	
Tumor size					
≤20 mm	3368 (56.7)	2046 (60.8)	0.001	822 (46.2)	<0.001
20–50 mm	2499 (42.1)	1282 (38.1)		921 (51.7)	
>50 mm	69 (1.2)	36 (1.1)		37 (2.1)	

**Table 3:** Characteristics of TNBC patients with three HER2 breast cancer subtypes.

Variables	Overall	HER2-0	HER2-Low	*p*
	1698	1059	639	
Age				
≤50	715 (42.1)	492 (46.5)	223 (34.9)	<0.001
>50	983 (57.9)	567 (53.5)	416 (65.1)	
Pathology Type				
IDC+ILC	1594 (93.9)	1006 (95.0)	588 (92.0)	<0.001
DCIS	74 (4.4)	29 (2.7)	45 (7.0)	
Others	30 (1.8)	24 (2.3)	6 (0.9)	
pN stage				
N0	1207 (71.1)	767 (72.4)	440 (68.9)	0.27
N1	319 (18.8)	191 (18.0)	128 (20.0)	
N2	105 (6.2)	58 (5.5)	47 (7.4)	
N3	67 (3.9)	43 (4.1)	24 (3.8)	
Ki-67				
≤14%	175 (10.3)	72 (6.8)	103 (16.1)	<0.001
>14%	1523 (89.7)	987 (93.2)	536 (83.9)	
Tumor size				
≤20 mm	780 (45.9)	487 (46.0)	293 (45.9)	0.845
20–50 mm	875 (51.5)	547 (51.7)	328 (51.3)	
>50 mm	43 (2.5)	25 (2.4)	18 (2.8)	

## Data Availability

The data presented in this study are not publicly available due to strict ethical restrictions and patient privacy protection requirements in accordance with the Institutional Review Board guidelines of Harbin Medical University Cancer Hospital. The aggregated and de-identified summary data supporting the findings of this study are included in the article and its Supplementary Materials. Further inquiries for the de-identified aggregated data can be directed to the corresponding author upon reasonable academic request.

## Supplementary Materials

The following supporting information can be downloaded at: https://www.mdpi.com/article/10.3390/cancers18101637/s1. Figure S1: Incidence of HER2-low breast cancers; Figure S2: HER2 subtypes distribution in the HR+ and TNBC; Figure S3: Incidence of HER2-low in the HR+ and TNBC subtypes; Figure S4: The survival of HER2-0, HER2-low and HER2-high; Table S1: Characteristics of IDC and ILC with three HER2 subtypes; Table S2: Characteristics of DCIS with three HER2 subtypes; Table S3: Characteristics of HR+ IDC and ILC with three HER2 subtypes; Table S4: Characteristics of HR+ DCIS with three HER2 subtypes; Table S5: Characteristics of all patients with IHC-1+ and IHC-2+ in HER2-low breast cancer; Table S6: Characteristics of HR+ patients with IHC-1+ and IHC-2+; Table S7: Characteristics of TNBC patients with IHC-1+ and IHC-2+; Table S8: Characteristics according to HER2 discordance status; Table S9: The pCR of HER2-0, HER2-low and HER2-high breast cancer after NACT; Table S10: The pCR in the HR+ and TNBC subtypes; Table S11: The pCR of HER2-low breast cancer; Table S12: Univariate analysis of npCR in the neoadjuvant chemotherapy patients; Table S13: Univariate analysis of bpCR in the neoadjuvant chemotherapy patients; Table S14: Univariate analysis of tpCR in the neoadjuvant chemotherapy patients; Table S15: The survival of HER2-low breast cancer.
