# Supplementary material for: Clinicopathological Characteristics, Prognosis, and Survival of HER2-Low Breast Cancer Patients Based on a Retrospective Cohort Study of 14,642 Patients
Source: Cancers (Basel). 2026 May 19;18(10):1637. doi: 10.3390/cancers18101637 (PMC13204811; doi:10.3390/cancers18101637)

Supplementary figure S1. Incidence of HER2-low breast cancers.

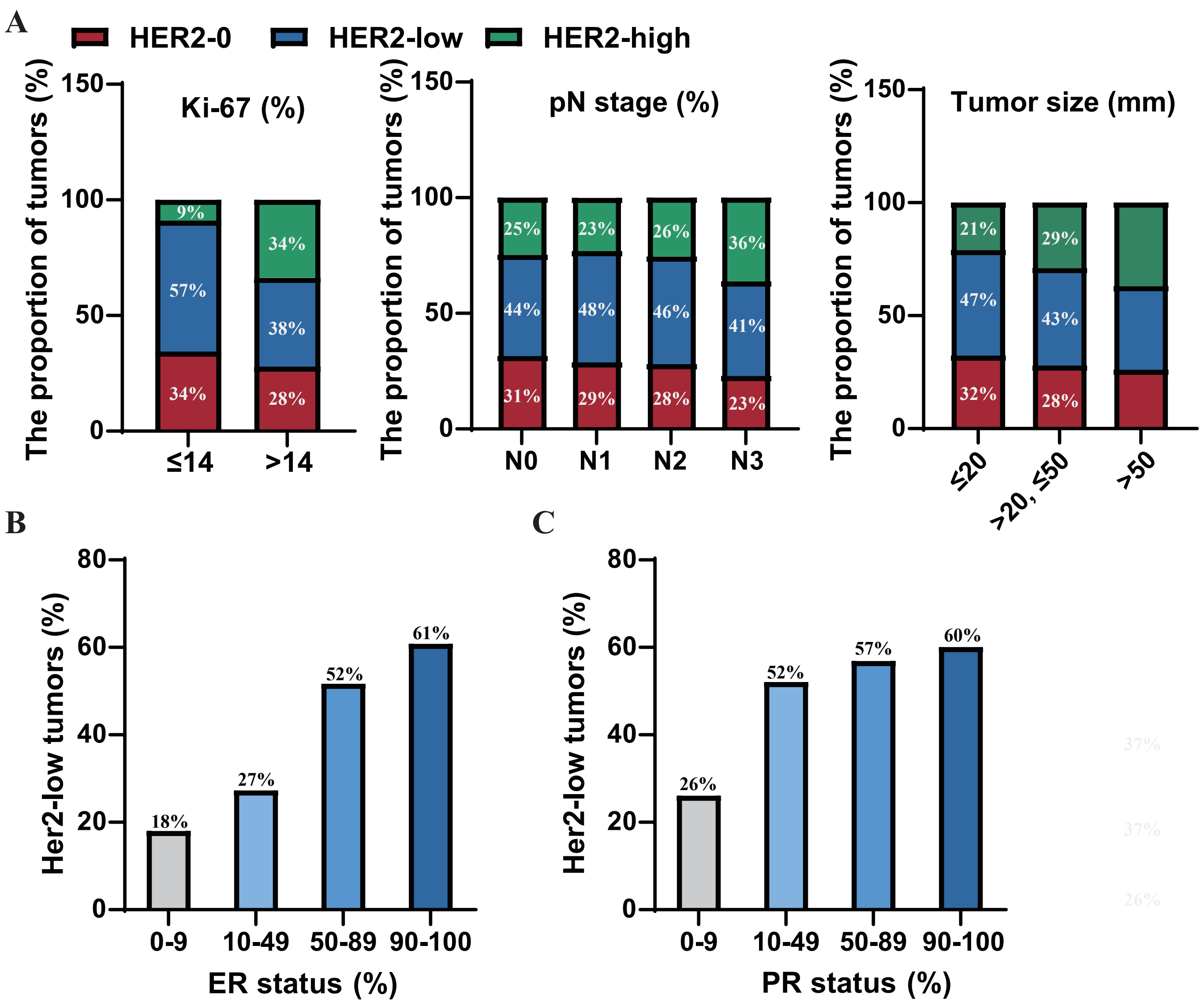

Supplementary figure S2. HER2 subtypes distribution in the HR+ and TNBC.

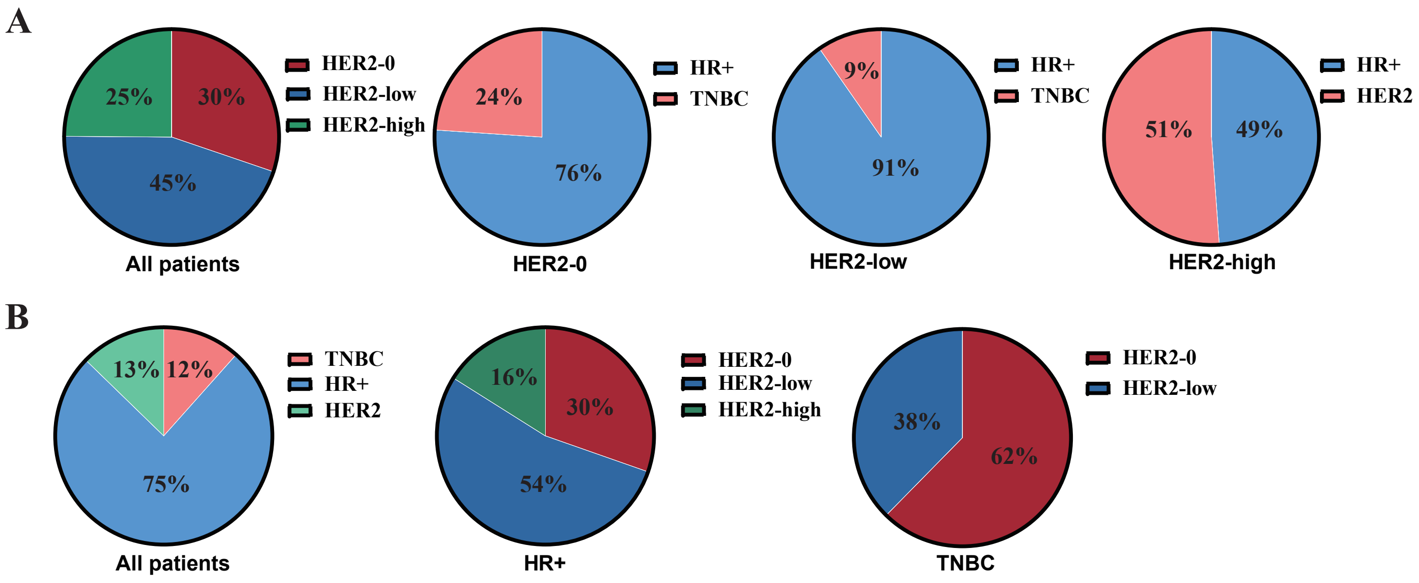

Supplementary figure S3. Incidence of HER2-low in the HR+ and TNBC subtypes.

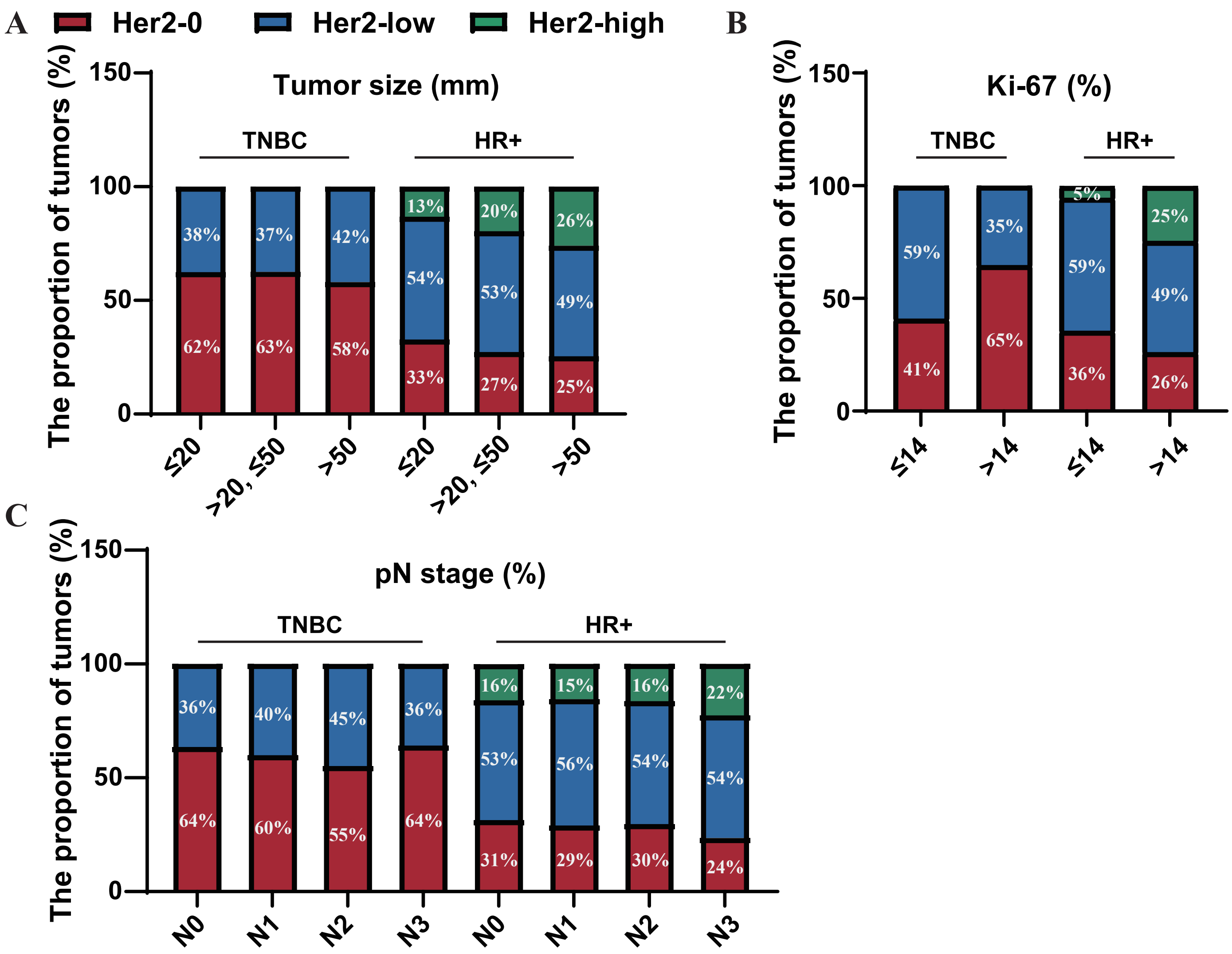

Supplementary figure S4. The survival of HER2-0, HER2-low and HER2-high.

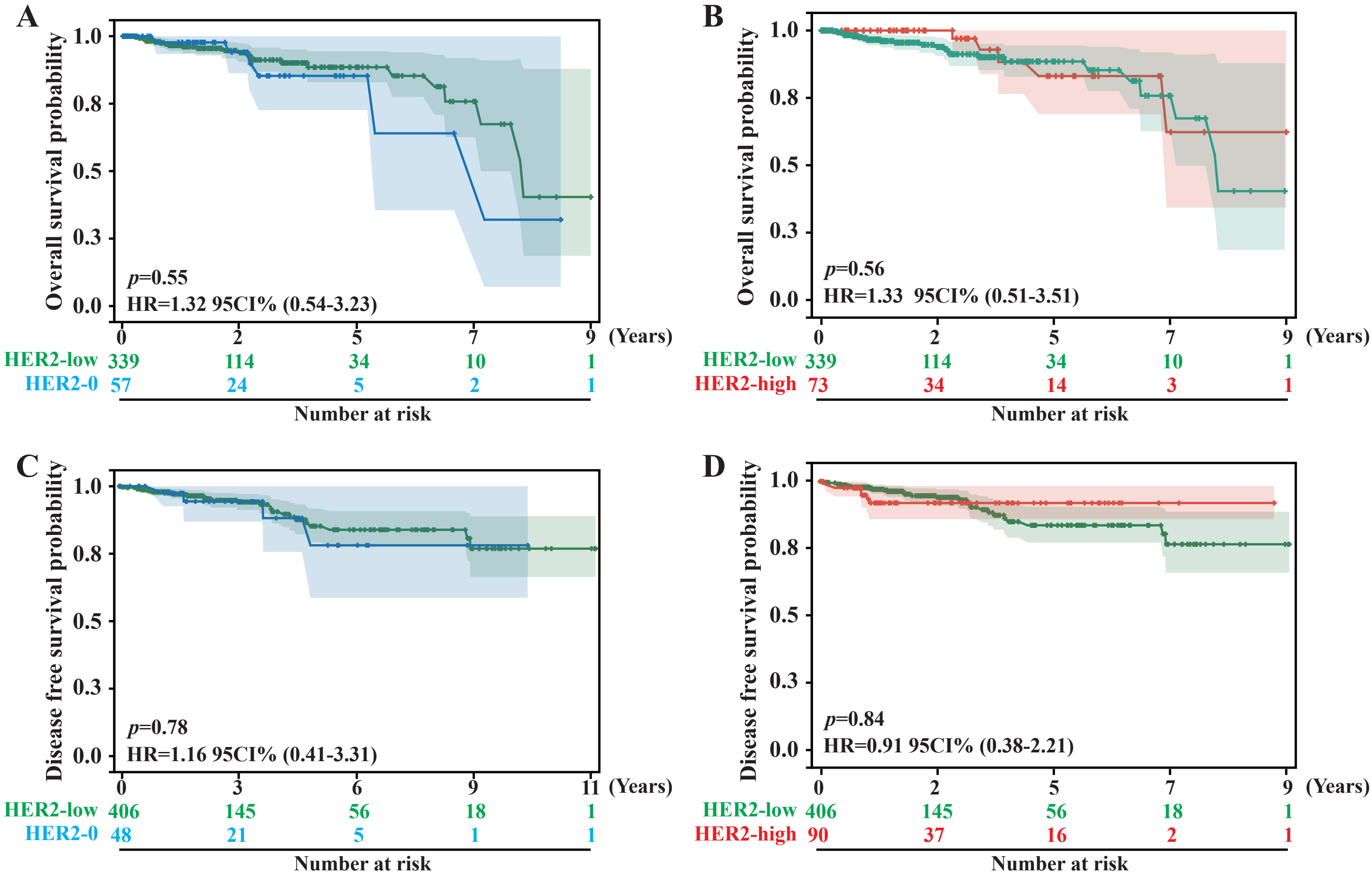

Supplement: Supplementary file 1 [file cancers-18-01637-s001.zip › Supplementary figure.pdf]
