# Supplementary material for: Clinicopathological Characteristics, Prognosis, and Survival of HER2-Low Breast Cancer Patients Based on a Retrospective Cohort Study of 14,642 Patients
Source: Cancers (Basel). 2026 May 19;18(10):1637. doi: 10.3390/cancers18101637 (PMC13204811; doi:10.3390/cancers18101637)
Supplement: Supplementary file 1 [file cancers-18-01637-s001.zip › Supplementary table.pdf]

Supplementary table S1. Characteristics of IDC and ILC with three HER2 subtypes.

Variables	HER2-Low	HER2-0	*P*	HER2-high	*P*
	**6100**	**4064**		**3044**	
**Age**					
≦50	2795 (45.8)	1936 (47.6)	0.075	1314 (43.2)	0.017
>50	3305 (54.2)	2128 (52.4)		1730 (56.8)	
**Histological Grade**					
I	230 (3.8)	149 (3.7)	<0.001	4 (0.1)	<0.001
II	4943 (81.0)	2830 (69.6)		1784 (58.6)	
III	927 (15.2)	1085 (26.7)		1256 (41.3)	
**pN stage**					
N0	3605 (59.1)	2582 (63.5)	<0.001	1699 (55.8)	<0.001
N1	1637 (26.8)	977 (24.0)		782 (25.7)	
N2	586 (9.6)	353 (8.7)		323 (10.6)	
N3	272 (4.5)	152 (3.7)		240 (7.9)	
**ER status**					
Negative	624 (10.2)	1063 (26.2)	<0.001	1551 (51.0)	<0.001
Positive	5476 (89.8)	3001 (73.8)		1493 (49.0)	
**PR status**					
Negative	977 (16.0)	1251 (30.8)	<0.001	1860 (61.1)	<0.001
Positive	5123 (84.0)	2813 (69.2)		1184 (38.9)	
**Ki-67**					
≦14%	2613 (42.8)	1539 (37.9)	<0.001	302 (9.9)	<0.001
>14%	3487 (57.2)	2525 (62.1)		2742 (90.1)	
**Tumor size**					
≦20mm	3362 (55.1)	2302 (56.6)	0.299	1343 (44.1)	<0.001
20-50mm	2651 (43.5)	1709 (42.1)		1635 (53.7)	
>50mm	87 (1.4)	53 (1.3)		66 (2.2)	

Supplementary table S2. Characteristics of DCIS with three HER2 subtypes.

Variables	HER2-Low	HER2-0	*P*	HER2-high	*P*
	454	323		589	
**Age**					
≦50	247 (54.4)	160 (49.5)	0.205	287 (48.7)	0.079
>50	207 (45.6)	163 (50.5)		302 (51.3)	
**Histological Grade**					
Low	136 (30.0)	143 (44.3)	<0.001	8 (1.4)	<0.001
Medium	136 (30.0)	72 (22.3)		38 (6.5)	
High	182 (40.1)	108 (33.4)		543 (92.2)	
**ER**					
Negative	46 (10.1)	30 (9.3)	0.789	388 (65.9)	<0.001
Positive	408 (89.9)	293 (90.7)		201 (34.1)	
**PR**					
Negative	68 (15.0)	49 (15.2)	1	452 (76.7)	<0.001
Positive	386 (85.0)	274 (84.8)		137 (23.3)	
**Ki-67**					
≦14%	335 (73.8)	250 (77.4)	0.287	169 (28.7)	<0.001
>14%	119 (26.2)	73 (22.6)		420 (71.3)	
**Tumor size**					
≦20mm	292 (64.3)	212 (65.6)	0.705	298 (50.6)	<0.001
>20mm	162 (35.7)	111 (34.4)		291 (49.4)	

Supplementary table S3. Characteristics of HR+ IDC and ILC with three HER2 subtypes.

Variables	HER2-Low	HER2-0	*P*	HER2-high	*P*
	5512	3058		1566	
**Age**					
≦50	2586 (46.9)	1464 (47.9)	0.407	800 (51.1)	0.004
>50	2926 (53.1)	1594 (52.1)		766 (48.9)	
**Histological Grade**					
I	229 (4.2)	146 (4.8)	0.177	1 (0.1)	<0.001
II	4705 (85.4)	2566 (83.9)		1081 (69.0)	
III	578 (10.5)	346 (11.3)		484 (30.9)	
**pN stage**					
N0	3214 (58.3)	1864 (61.0)	0.041	877 (56.0)	0.005
N1	1510 (27.4)	790 (25.8)		421 (26.9)	
N2	540 (9.8)	295 (9.6)		164 (10.5)	
N3	248 (4.5)	109 (3.6)		104 (6.6)	
**Ki-67**					
≦14%	2533 (46.0)	1482 (48.5)	0.027	187 (11.9)	<0.001
>14%	2979 (54.0)	1576 (51.5)		1379 (88.1)	
**Tumor size**					
≦20mm	3090 (56.1)	1845 (60.3)	<0.001	701 (44.8)	<0.001
20-50mm	2353 (42.7)	1185 (38.8)		833 (53.2)	
>50mm	69 (1.3)	28 (0.9)		32 (2.0)	

Supplementary table S4. Characteristics of HR+ DCIS with three HER2 subtypes.

Variables	HER2-Low	HER2-0	*P*	HER2-high	*P*
	**409**	**294**		**214**	
**Age**					
≦50	238 (58.2)	149 (50.7)	0.058	129 (60.3)	0.676
>50	171 (41.8)	145 (49.3)		85 (39.7)	
**Histological Grade**					
Low	131 (32.0)	139 (47.3)	<0.001	6 (2.8)	<0.001
Medium	134 (32.8)	70 (23.8)		26 (12.1)	
High	144 (35.2)	85 (28.9)		182 (85.0)	
**Ki-67**					
≦14%	313 (76.5)	237 (80.6)	0.229	66 (30.8)	<0.001
>14%	96 (23.5)	57 (19.4)		148 (69.2)	
**Tumor size**					
≦20mm	273 (66.7)	194 (66.0)	0.833	121 (56.5)	0.012
>20mm	136 (33.3)	100 (34.0)		93 (43.4)	

Supplementary table S5 Characteristics of all patients with IHC-1+ and IHC-2+ in HER2-low breast cancer.

**Variables**	**Overall**	**IHC-1+**	**IHC-2+**	***p***
**n**	**n=6575**	**5446**	**1129**	
**Age**				
≦50	3052 (46.4)	2523 (46.3)	529 (46.9)	0.771
>50	3523 (53.6)	2923 (53.7)	600 (53.1)	
**Pathology Type**				
IDC+ILC	6100 (92.8)	4982 (91.5)	1118 (99.0)	<0.001
DCIS	454 (6.9)	447 (8.2)	7 (0.6)	
Others	21 (0.3)	17 (0.3)	4 (0.4)	
**pN stage**				
N0	4074 (62.0)	3383 (62.1)	691 (61.2)	0.598
N1	1639 (24.9)	1363 (25.0)	276 (24.4)	
N2	590 (9.0)	480 (8.8)	110 (9.7)	
N3	272 (4.1)	220 (4.0)	52 (4.6)	
**ER status**				
Negative	676 (10.3)	558 (10.2)	118 (10.5)	0.878
Positive	5899 (89.7)	4888 (89.8)	1011 (89.5)	
**PR status**				
Negative	1055 (16.0)	861 (15.8)	194 (17.2)	0.271
Positive	5520 (84.0)	4585 (84.2)	935 (82.8)	
**Ki-67**				
≦14%	2955 (44.9)	2566 (47.1)	389 (34.5)	<0.001
>14%	3620 (55.1)	2880 (52.9)	740 (65.5)	
**Tumor size**				
≦20mm	3661 (55.7)	3088 (56.7)	573 (50.8)	0.001
20-50mm	2827 (43.0)	2284 (41.9)	543 (48.1)	
>50mm	87 (1.3)	74 (1.4)	13 (1.2)	

Supplementary table S6. Characteristics of HR+ patients with IHC-1+ and IHC-2+.

**Variables**	**Overall**	**IHC-1+**	**IHC-2+**	***p***
**n**	**n=5936**	**4923**	**1013**	
**Age**				
≦50	2829 (47.7)	2331 (47.3)	498 (49.2)	0.309
>50	3107 (52.3)	2592 (52.7)	515 (50.8)	
**Pathology Type**				
IDC+ILC	5512 (92.9)	4508 (91.6)	1004 (99.1)	<0.001
DCIS	409 (6.9)	403 (8.2)	6 (0.6)	
Others	15 (0.3)	12 (0.2)	3 (0.3)	
**pN stage**				
N0	3634 (61.2)	3015 (61.2)	619 (61.1)	0.491
N1	1511 (25.5)	1265 (25.7)	246 (24.3)	
N2	543 (9.1)	439 (8.9)	104 (10.3)	
N3	248 (4.2)	204 (4.1)	44 (4.3)	
**ER status**				
Negative	37 (0.6)	35 (0.7)	2 (0.2)	0.095
Positive	5899 (99.4)	4888 (99.3)	1011 (99.8)	
**PR status**				
Negative	416 (7.0)	338 (6.9)	78 (7.7)	0.379
Positive	5520 (93.0)	4585 (93.1)	935 (92.3)	
**Ki-67**				
≦14%	2852 (48.0)	2479 (50.4)	373 (36.8)	<0.001
>14%	3084 (52.0)	2444 (49.6)	640 (63.2)	
**Tumor size**				
≦20mm	3368 (56.7)	2839 (57.7)	529 (52.2)	0.004
20-50mm	2499 (42.1)	2025 (41.1)	474 (46.8)	
>50mm	69 (1.2)	59 (1.2)	10 (1.0)	

Supplementary table S7. Characteristics of TNBC patients with IHC-1+ and IHC-2+.

Variables	Overall	IHC-1+	IHC-2+	*p*
n	639	523	116	
**Age**				
≦50	223(34.9)	223(34.9)	223(34.9)	0.053
>50	416(65.1)	331(63.3)	85(73.3)	
**Pathology Type**				
IDC+ILC	588(92.0)	474(90.6)	114(98.3)	0.016
DCIS	45(7.0)	44(8.4)	1(0.9)	
Others	6(0.9)	5(1.0)	1(0.9)	
**pN stage**				
N0	440(68.9)	368(70.4)	72(62.1)	0.046
N1	128(20.0)	98(18.7)	30(25.9)	
N2	47(7.4)	41(7.8)	6(25.9)	
N3	24(3.8)	16(3.1)	8(6.9)	
**Ki-67**				
≦14%	103(16.1)	87(16.6)	16(13.8)	0.54
>14%	536(83.9)	436(83.4)	100(86.2)	
**Tumor size**				
≦20mm	293(45.9)	249(47.6)	44(37.9)	0.149
20-50mm	328(51.3)	259(49.5)	69(59.5)	
>50mm	18(2.8)	15(2.9)	3(2.6)	

Supplementary table S8. Characteristics according to HER2 discordance status.

		Total n=1526	HER2 discordance status		*p*
			**HER2 concordant n=874**	**HER2 discordant n=652**	
**Age (%)**	**≤50**	772	434 (56.22)	338 (43.78)	0.428
	**＞50**	754	440 (58.36)	314 (41.64)	
**Ki67 (%)**	**≤14%**	394	205 (52.03)	189 (47.97)	0.017
	**＞14%**	1132	669 (59.10)	463 (40.9)	
**Pathology (%)**	**Invasive ductal carcinoma**	1258	704 (55.96)	554 (44.04)	0.017
	**Invasive lobular carcinoma**	217	132 (60.83)	85 (39.17)	
	**Others**	51	38 (74.51)	13 (25.49)	
**Histological (%)**	**I**	100	46 (46.00)	54 (54.00)	0.001
	**II**	1177	663 (56.33)	514 (43.67)	
	**III**	249	165 (66.27)	84 (33.73)	
**Swollen lymph nodes (%)**	**Negative**	543	330 (60.77)	213 (39.23)	0.046
	**Positive**	983	544 (55.34)	439 (44.66)	
**T (%)**	**≤2cm**	245	149 (60.82)	96 (39.18)	0.316
	**＞2cm, ≤5cm**	1074	602 (56.05)	472 (43.95)	
	**＞5cm**	207	123 (59.42)	84 (40.58)	

Supplementary table S9 The pCR of HER2-0, HER2-low and HER2-high breast cancer after NACT.

	**bpCR**	**non-bpCR**	**Total**	***P***
**HER2-Low**	79 (17.7)	368 (82.3)	447	
**HER2-0**	53 (14.6)	311 (85.4)	364	0.232
**HER2-high**	38 (16.0)	199 (84.0)	237	0.588
**Total**	170	878	1048	

	**npCR**	**non-npCR**	**Total**	***P***
**HER2-Low**	129 (38.9)	203 (61.1)	332	
**HER2-0**	106 (38.5)	169 (61.5)	275	0.938
**HER2-high**	56 (34.4)	107(65.6)	163	0.331
**Total**	291	479	770	

	**tpCR**	**non-tpCR**	**Total**	***P***
**HER2-Low**	35 (10.4)	303 (89.6)	338	
**HER2-0**	19 (7.4)	239 (92.6)	258	0.208
**HER2-high**	14 (10.5)	119 (89.5)	133	0.956
**Total**	68	661	729	

Supplementary table S10. The pCR in the HR+ and TNBC subtypes.

	bpCR	non-bpCR	Total	*P*
**HER2-Low**	69 (17.1)	335 (82.9)	404	
**HER2-0**	35 (13.3)	229 (86.7)	264	0.183
**HER2-high**	15 (16.7)	75 (83.3)	90	0.925
**Total**	119	639	758	

	npCR	non-npCR	Total	*P*
**HER2-Low**	116 (37.3)	195 (62.7)	311	
**HER2-0**	81 (38.8)	128 (61.2)	209	0.737
**HER2-high**	18 (30.0)	42 (70.0)	60	0.281
**Total**	215	365	580	

	tpCR	non-tpCR	Total	*P*
**HER2-Low**	30 (10.8)	247 (89.2)	277	
**HER2-0**	14 (7.1)	182 (92.9)	196	0.174
**HER2-high**	4 (5.2)	73 (94.8)	77	0.189
**Total**	48	502	550	

	bpCR	non-bpCR	Total	*P*
**HER2-0**	18 (18.0)	82 (82.0)	100	1
**HER2-Low**	12 (18.8)	52 (81.2)	64	
**Total**	30	134	164	

	npCR	non-npCR	Total	*P*
**HER2-0**	25 (37.9)	41 (62.1)	66	0.84
**HER2-Low**	16 (40.0)	24 (60.0)	40	
**Total**	41	65	106	

	tpCR	non-tpCR	Total	*P*
**HER2-0**	5 (8.1)	57 (91.9)	62	0.494
**HER2-Low**	5 (13.5)	32 (86.5)	37	
**Total**	10	89	99	

Supplementary table S11 The pCR of HER2-low breast cancer.

Study	Year	Design	No. of patients received NAC	pCR: HER2-low vs. HER2-0		
				**Total**	**HR-positive**	**TNBC**
Guansheng Zhong et al.[16]	2023	The National Cancer Database	41500	27.4% vs. 35.5%, *p*＜0.001	17.7% vs. 20.6%, *p*＜0.001	46.6% vs. 48.8%, *p*＜0.001
Youzhao Ma et al. [26]	2022	Retrospective Analysis	690	14.2% vs.23.0% , *p*=0.005	7.8% vs.12.4% *p*=0.18	30.7% vs.31.8%, *p*=0.85
Guochun Zhang et al. [18]	2022	Retrospective Analysis	523	15.9% vs. 37.5%, *p*=0.042	9.3% vs. 20.0%, *p*=0.358	55.6%vs. 66.7%, *p*=1
Yingbo Shao et al.20[4]	2022	Retrospective Analysis	314	24.3% vs. 36.4%, *p*=0.032	18.7% vs. 32.1%, *p*=0.035	41.8% vs. 43.8%, *p*=0.860
Shichao Zhang et al. [38]	2023	Retrospective Analysis	3070	—	17.3% vs. 13.7%, *p*=0.016	—
Weiqiang Qiao et al.[27]	2023	Retrospective Analysis	132	20.0% vs. 37.1%, *p*=0.03	15.1% vs21.7%, *p*=0.7	35.3% vs. 46.2%, *p*=0.45
Silvia Mihaela Ilie et al. [30]	2023	Retrospective Analysis	111	—	4% vs. 6%, *p*=0.3	35% vs. 47%, *p*=0.088
Weiwei Xu et al. [28]	2022	Retrospective Analysis	429	tpCR (5.2% vs. 14.2%, *p*=0.002) bpCR (6.4% vs.17.3%, *p*=0.001) npCR (26.3% vs. 37.7%, *p*=0.014)	tpCR (3.4% vs. 2.5%, *p*=0.695) bpCR (3.9% vs. 3.7%, *p*=0.955) npCR (21.6% vs. 21.0%, *p*=0.904)	tpCR (11.9% vs. 25.9%, *p*=0.04) bpCR (15.3% vs. 30.9%, *p*=0.033) npCR (43.1% vs. 54.3%, *p*=0.193)
Alexndre de Nonneville et	2022	Retrospective Analysis	1111	23% vs. 30%, *p*=0.013	10% vs 16%, *p*=0.046	42% vs 46%, *p*=0.356

al. [29]						
Carsten Denkert et al.[13]	2021	four prospective clinical trials	2310	29.2% vs. 39.0% , *p*=0.0002	17.5% vs. 23.6% *p*=0.024	50.1% vs. 48.0%, *p*=0.21
Camille Domergue et al.[31]	2022	Retrospective Analysis	437	—	—	35.7% vs. 41.8%, *p*=0.284
Luciana de Moura Leite et al. [32]	2021	Retrospective Analysis	855	—	13% vs. 9.5%, *p*=0.27	51% vs. 47%, *p*=0.64
George Douganiotis et al. [33]	2022	Retrospective Analysis	87	8.8% vs. 9.1% , *p*=0.954	—	—
Fátima R. Alves et al. [34]	2022	Retrospective Analysis	72	14.6% vs. 29.0% , *p*=0.15	14.0% vs. 27.0% *p*=0.36	17.0% vs. 30.0%, *p*=0.67

Supplementary table S12. Univariate analysis of npCR in the neoadjuvant chemotherapy patients.

Variables	Overall	nPCR, No.(%)	non-nPCR, No.(%)	*P*
	770	291	479	
**Age**				
≤50	374 (48.6)	155 (41.4)	219 (58.6)	0.05
＞50	396 (51.4)	136 (34.3)	260 (65.7)	
**ER status**				
Negative	204 (26.5)	80 (39.2)	124 (60.8)	0.686
Positive	566 (73.5)	211 (37.3)	355 (62.7)	
**PR status**				
Negative	287 (37.3)	111 (38.7)	176 (61.3)	0.754
Positive	483 (62.7)	180 (37.3)	303 (62.7)	
**Ki-67**				
≤14%	205 (26.6)	90 (43.9)	115 (56.1)	0.043
＞14%	565 (73.4)	201 (35.6)	364 (64.4)	
**Pathology Type**				
IDC	645 (83.8)	250 (38.8)	395 (61.2)	0.003
ILC	101 (13.1)	40 (39.6)	61 (60.4)	
Others	24 (3.1)	1 (4.2)	23 (95.8)	
**Histological Grade**				
I	50 (6.5)	16 (32.0)	34 (68.0)	0.455
II	598 (77.7)	224 (37.5)	374 (62.5)	
III	122 (15.8)	51 (41.8)	71 (58.2)	
**Tumor size**				
≤ 2cm	123 (16.0)	52 (42.3)	71 (57.7)	0.369
＞2, ≤5cm	536 (69.6)	202 (37.7)	334 (62.3)	
＞5cm	111 (14.4)	37 (33.3)	74 (66.7)	
**HER2 status**				
0	275 (35.7)	106 (38.5)	169 (61.5)	0.8
1+	261 (33.9)	102 (39.1)	159 (60.9)	
2+	95 (12.3)	32 (33.7)	63 (66.3)	
3+	139 (18.1)	51 (36.7)	88 (63.3)	

Supplementary table S13. Univariate analysis of bpCR in the neoadjuvant chemotherapy patients.

Variables	Overall	bPCR, No.(%)	non-bPCR, No.(%)	*P*
	**1048**	**170**	**878**	
**Age**				
≤50	518 (49.4)	89 (17.2)	429 (82.8)	0.453
＞50	530 (50.6)	81 (15.3)	449 (84.7)	
**ER status**				
Negative	313 (29.9)	57 (18.2)	256 (81.8)	0.294
Positive	735 (70.1)	113 (15.4)	622 (84.6)	
**PR status**				
Negative	424 (40.5)	73 (17.2)	351 (82.8)	0.525
Positive	624 (59.5)	97 (15.5)	527 (84.5)	
**Ki-67**				
≤14%	262 (25.0)	44 (16.8)	218 (83.2)	0.847
＞14%	786 (75.0)	126 (16.0)	660 (84.0)	
**Pathology Type**				
IDC	852 (81.3)	139 (16.3)	713 (83.7)	0.959
ILC	161 (15.4)	25 (15.5)	136 (84.5)	
Others	35 (3.3)	6 (17.1)	29 (82.9)	
**Histological Grade**				
I	64 (6.1)	7 (10.9)	57 (89.1)	0.495
II	817 (78.0)	135 (16.5)	682 (83.5)	
III	167 (15.9)	28 (16.8)	139 (83.2)	
**Tumor size**				
≤ 2cm	152 (14.5)	42 (27.6)	110 (72.4)	<0.001
＞2, ≤5cm	750 (71.6)	100 (13.3)	650 (86.7)	
＞5cm	146 (13.9)	28 (19.2)	118 (80.8)	
**HER2 status**				
0	364 (34.7)	53 (14.6)	311 (85.4)	0.704
1+	342 (32.6)	58 (17.0)	284 (83.0)	
2+	135 (12.9)	25 (18.5)	110 (81.5)	
3+	207 (19.8)	34 (16.4)	173 (83.6)	

Supplementary table S14. Univariate analysis of tpCR in the neoadjuvant chemotherapy patients.

**Variables**	**Overall**	**tPCR, No.(%)**	**non-tPCR, No.(%)**	***P***
	**729**	**68**	**661**	
**Age**				
≤50	353 (48.4)	39 (11.0)	314 (89.0)	0.156
＞50	376 (51.6)	29 (7.7)	347 (92.3)	
**ER status**				
Negative	193 (26.5)	22 (11.4)	171 (88.6)	0.313
Positive	536 (73.5)	46 (8.6)	490 (91.4)	
**PR status**				
Negative	267 (36.6)	27 (10.1)	240 (89.9)	0.673
Positive	462 (63.4)	41 (8.9)	421 (91.1)	
**Ki-67**				
≤14%	194 (26.6)	22 (11.3)	172 (88.70)	0.327
＞14%	535 (73.4)	46 (8.6)	489 (91.4)	
**Pathology Type**				
IDC	614 (84.2)	56 (9.1)	558 (90.9)	0.483
ILC	92 (12.6)	11 (12.0)	81 (88.0)	
Others	23 (3.2)	1 (4.3)	22 (95.7)	
**Histological Grade**				
I	46 (6.3)	2 (4.3)	44 (95.7)	0.483
II	573 (78.6)	55 (9.6)	518 (90.4)	
III	110 (15.1)	11 (10.0)	99 (90.0)	
**Tumor size**				
≤ 2cm	107 (14.7)	20 (18.7)	87 (81.3)	0.001
＞2, ≤5cm	515 (70.6)	36 (7.0)	479 (93.0)	
＞5cm	107 (14.7)	12 (11.2)	95 (88.8)	
**HER2 status**				
0	258 (35.4)	19 (7.4)	239 (92.6)	0.439
1+	249 (34.2)	28 (11.2)	221 (88.8)	
2+	89 (12.2)	7 (7.9)	82 (92.1)	
3+	133 (18.2)	14 (10.5)	119 (89.5)	

Supplementary table S15 The survival of HER2-low breast cancer.

Study	Year	Design	No. of patients received NAC	survival: HER2-low vs. HER2-0		
				**Total**	**HR-positive**	**TNBC**
Guansheng Zhong et al.[16]	2023	The National Cancer Database	41500	OS, $p<0.001$	OS, $p=0.005$	OS, $p=0.004$
Youzhao Ma et al. [26]	2022	Retrospective Analysis	690	DFS, $p=0.846$	—	—
Guochun Zhang et al. [18]	2022	Retrospective Analysis	523	DFS, $p=0.271$	—	—
Yingbo Shao et al.20[4]	2022	Retrospective Analysis	314	OS, $p=0.258$; DFS, $p=0.332$	OS, $p=0.819$; DFS, $p=0.633$	OS, $p=0.200$; DFS, $p=0.319$
Shichao Zhang et al. [38]	2023	Retrospective Analysis	3070	OS, $p=0.037$; DFS, $p=0.610$	—	—
Weiqiang Qiao et al.[27]	2023	Retrospective Analysis	132	OS, p=0.016; DFS, p=0.023	OS, $p=0.007$; DFS, $p=0.006$	OS, $p=0.968$; DFS, $p=0.997$
Silvia Mihaela Ilie et al. [30]	2023	Retrospective Analysis	111	—	OS, p=0.717; RFS, p=0.429	OS, $p=0.028$; RFS, $p=0.0435$
Weiwei Xu et al. [28]	2022	Retrospective Analysis	429	DFS, $p=0.816$	DFS, $p=0.530$	DFS, $p=0.362$

Alexndre de Nonneville et al. [29]	2022	Retrospective Analysis	1111	DFS, p=0.742	DFS, p=0.511	DFS, p=0.622
Carsten Denkert et al.[13]	2021	four prospective clinical trials	2310	OS, p=0.0016; DFS, p=0.0084	OS, p=0.13; DFS, p=0.39	OS, p=0.016; DFS, p=0.0076
Camille Domergue et al.[31]	2022	Retrospective Analysis	437	OS, p=0.25; DFS, p=0.362	—	—
Luciana de Moura Leite et al. [32]	2021	Retrospective Analysis	855	—	OS, p=0.11; RFS, p=0.47	OS, p=0.71; DFS, p=0.23
George Douganiotis et al. [33]	2022	Retrospective Analysis	87	RFS, $p>0.05$	—	—
Fátima R. Alves et al. [34]	2022	Retrospective Analysis	72	OS, p=0.35; DFS, p=0.97	—	—
Bin Xu et al. [17]	2023	Retrospective Analysis	2605	—	OS, p=0.805; DFS, p=0.688	OS, p=0.085; DFS, p=0.418
Nanae Horisawa et al. [19]	2021	Retrospective Analysis	4918	—	OS, p=0.215; DFS, p=0.151	OS, p=0.152; DFS, p=0.306
Wei Wang et al. [35]	2022	Retrospective Analysis	148	DFS, p=0.04	—	—
Sora Kang et al. [36]	2023	Retrospective Analysis	1572	OS, $p<0.001$; DFS, $p<0.001$	OS, p=0.064; DFS, p=0.053	OS, p=0.29; DFS, p=0.12

Chang-Gen Liu et al. [37]	2023	Retrospective Analysis	4598	OS, p=0.437	OS, p=0.281	OS, p=0.947
